# Comparison of the effects of sugammadex and neostigmine on hospital stay in robot-assisted laparoscopic prostatectomy: a retrospective study

**DOI:** 10.1186/s12871-020-01088-6

**Published:** 2020-07-21

**Authors:** Byung-Hun Min, Tak Kyu Oh, In-Ae Song, Young-Tae Jeon

**Affiliations:** 1 Department of Anesthesiology and Pain Medicine, Seoul National University Bundang Hospital, Gumi-ro 173 Beon-gil, Bundang-gu, Seongnam-si, Gyeonggi-do Republic of Korea; 2 Interdepartment of Critical Care Medicine, Seoul National University Bundang Hospital, 166, Gumi-ro 173 Beon-gil, Bundang-gu, Seongnam-si, Gyeonggi-do 463-707 Republic of Korea; 3 Department of Anaesthesiology and Pain Medicine, Seoul National University, 103 Daehak-ro, Jongno-gu, Seoul, Republic of Korea

**Keywords:** Hospital length of stay, Neuromuscular blocking agents, Reversal agents, Rocuronium, Sugammadex

## Abstract

**Background:**

Sugammadex reduces postoperative complications. We sought to determine whether it could reduce the length of hospital stay, post-anesthetic recovery time, unplanned readmission, and charges for patients who underwent robot-assisted laparoscopic prostatectomy (RALP) when compared to neostigmine.

**Methods:**

This was a retrospective observational study of patients who underwent RALP between July 2012 and July 2017, in whom rocuronium was used as a neuromuscular blocker. The primary outcome was the length of hospital stay after surgery in patients who underwent reversal with sugammadex when compared to those who underwent reversal with neostigmine. The secondary outcomes were post-anesthetic recovery time, hospital charges, and unplanned readmission within 30 days after RALP.

**Results:**

In total, 1430 patients were enrolled. Using a generalized linear model in a propensity score-matched cohort, sugammadex use was associated with a 6% decrease in the length of hospital stay (mean: sugammadex 7.7 days vs. neostigmine 8.2 days; odds ratio [OR] 0.94, 95% confidence interval [CI] [0.89, 0.98], *P* = 0.008) and an 8% decrease in post-anesthetic recovery time (mean: sugammadex 36.7 min vs. neostigmine 40.2 min; OR 0.92, 95% CI [0.90, 0.94], *P* < 0.001) as compared to neostigmine use; however, it did not reduce the 30-day unplanned readmission rate (*P* = 0.288). The anesthesia charges were higher in the sugammadex group than in the neostigmine group (*P* < 0.001); however, there were no significant differences between the groups in terms of postoperative net charges (*P* = 0.061) and total charges (*P* = 0.100).

**Conclusions:**

Compared to the reversal of rocuronium effects with neostigmine, reversal with sugammadex after RALP was associated with a shorter hospital stay and post-anesthetic recovery time, and was not associated with 30-day unplanned readmission rates and net charges.

## Background

Sugammadex rapidly reverses the effects of neuromuscular blockade (NMB) from agents such as rocuronium or vecuronium. It is much more expensive than classical acetyl-cholinesterase inhibitors for the reversal of NMB (e.g., neostigmine). However, it can rapidly and definitively eliminate the effect of NMB, without causing side-effects due to muscarinic receptor activation [1]. Sugammadex could better reduce the hospital length of stay (LOS), and accelerate the postoperative discharge from the post-anesthesia care unit (PACU), compared to neostigmine in patients who underwent general abdominal surgery; however, it increased NMB and NMB reversal-related costs [2, 3]. Few studies have assessed the cost-effectiveness of sugammadex, and their results were controversial because healthcare systems differ markedly among countries [4]. For example, in a retrospective study in Italy, sugammadex eliminated postoperative residual curarization and saved costs related to residual NMB management [5]. In contrast, in an evidence-based review, there was little evidence of economic advantage for sugammadex use [4] . On the other hand, a previous study revealed that sugammadex reduced hospital LOS, 30-day unplanned readmission, and the hospital charge for patients undergoing major abdominal surgeries [6].

However, there has been no report on the effects of using sugammadex for robot-assisted laparoscopic prostatectomy (RALP), a costly but promising surgery that has a relatively lower complication risk and faster recovery than open retropubic surgery; however, it involves an operation lasting several hours in a very steep Trendelenburg position [7]. In a prolonged Trendelenburg position, the mean airway pressure increases following reduced vital capacity and forced expiratory volume1 at 5 days postoperatively [8]. Additionally, increased abdominal pressure causes pulmonary complications, such as aspiration or atelectasis [9]. Therefore, a rapid and proper reversal of NMB in RALP might reduce the hospital stay, post-anesthetic recovery time, readmission rate and charge by facilitating early mobilization and breathing exercises. We sought to determine whether sugammadex could reduce postoperative hospital LOS, post-anesthetic recovery time, hospital charges, and 30-day unplanned readmission in patients undergoing RALP.

## Methods

### Ethics approval and consent to participate

This study was approved by the Institutional Review Board (B-1901/514–115) of Seoul National University Bundang Hospital, which waived the requirement for obtaining informed patient consent.

### Study design, participants, and data collection

In this retrospective cohort study, all patient data were collected from electronic medical records. A medical informatics team extracted the medical records based on the patient selection criteria. Patients aged > 19 years who underwent elective RALP under general anesthesia between July 1, 2012, and July 31, 2017, were reviewed. We included patients who were administered only rocuronium. Patients who underwent combined surgeries (e.g., prostatectomy combined with nephrectomy), who were admitted to the intensive care unit without NMB reversal, or who had incomplete records were excluded.

Patients demographic characteristics (age, height, weight, body mass index), perioperative conditions (Charlson Comorbidity Index, American Society of Anesthesiologists physical status classification [ASA class], hospital LOS, 30-day unplanned readmission), and anesthesia and operative factors (types of sedatives, inhalational anesthetics, dose of remifentanil, and types and dose of NMB and reversal agents, duration of anesthesia, recovery time from anesthesia in PACU, estimated blood loss, and surgical proficiency) were reviewed.

### Anesthesia for RALP

RALP was performed under general anesthesia using inhalation anesthetics such as sevoflurane or desflurane or continuous propofol infusion with continuous intravenous remifentanil infusion. Propofol (1.5 mg kg^− 1^) was used to induce anesthesia when using inhalational anesthetics. Intravenous injection of a rocuronium bolus was used to initiate and maintain muscle relaxation under train-of-four (TOF) monitoring using a nerve stimulator. Neostigmine (0.02–0.05 mg kg^− 1^) or sugammadex (2 mg kg^− 1^) was used to reverse rocuronium. In all patients receiving neostigmine, glycopyrrolate was co-administered to prevent cholinergic complications.

### Clinical outcomes

The primary outcome was the difference in postoperative hospital LOS. Secondary outcomes were the post-anesthetic recovery time in the PACU, hospital charges and unplanned readmission within 30 days. The net hospital charge was defined as the total charge minus the charge of surgery and anesthesia. In South Korea, the national healthcare insurance covers two-thirds of the healthcare charge, and its coverage standard is updated regularly [10]. Data on unplanned hospital readmissions within 30 days of discharge after RALP were collected from electronic medical records. Patients who required readmissions for further evaluation and treatment of other underlying diseases were excluded.

### Statistical analysis

Categorical variables are presented as medians (25th/75th percentile) and numbers (%), whereas continuous variables are presented as mean (standard deviation) values. To adjust for confounding factors, we used the propensity score matching method without replacement, to balance covariates between groups. Age (> 65 years), Body mass index, Charlson Comorbidity Index score, ASA score (Classes 1, 2, and ≥ 3), intraoperative rocuronium,and remifentanil dosage, and total intravenous anesthesia (TIVA), duration of anesthesia (h), estimated blood loss (L), surgical proficiency (surgeons with experience in more than 200 cases [11]), and distance between home and hospital were matched as covariates in a 1:1 ratio between the groups, with a 0.3 caliper, by the nearest neighbor method. Sufficient covariate balance between the groups was determined by an absolute standardized difference ≤ 0.1. The MatchIt package of the R program (version 3.4.4; www.r-project.org) was used for propensity score-matching; the analysis was performed with SPSS software (IBM SPSS Statistics ver. 24; IBM Corp., Armonk, NY, USA).

After confirming balance in the matched cohort, generalized linear models with a logarithmic link function, with a Poisson distribution, were used to analyze correlations of NMB reversal agent with post-surgical hospital LOS and the post-anesthetic recovery time. Generalized linear models with a logarithmic link function with the gamma distribution were used to analyze the correlation between hospital charge and reversal agent. The association between the 30-day unplanned readmission rate and reversal agent was analyzed using binary logistic regression analysis.

*P*-values < 0.05 were considered statistically significant.

## Results

**Fig. 1:**
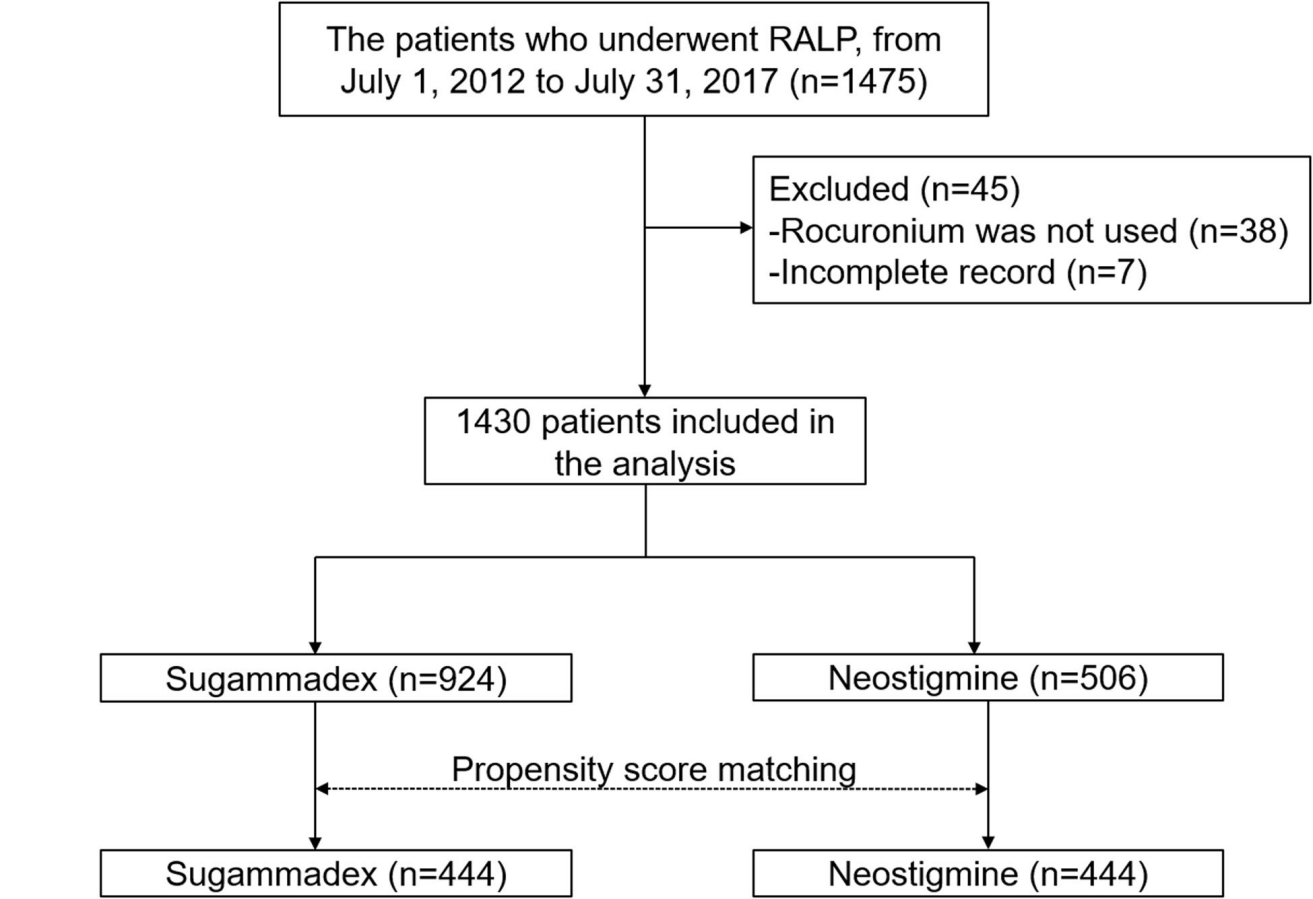
Flow chart of patient selection. Medical records of patients who underwent RALP between July 1, 2012 and July 31, 2017 were reviewed, and 1475 patients were initially included in the analysis; 38 were excluded owing to cisatracurium use, and 7 were excluded due to incomplete medical records. Finally, 1430 patients were included in the study; after propensity score matching, 444 were allocated in each group, namely, sugammadex and neostigmine

This study eventually included 1430 patients. In total, 1475 patients underwent elective RALP from July 1, 2012, to July 31, 2017; of these, 45 were excluded because rocuronium was not used intraoperatively (*n* = 38) or medical records were incomplete (*n* = 7). Sugammadex was used in 924 (64.6%), and neostigmine was used in 506 (35.4%) patients in this study (Fig. 1).

Patient demographics and clinical outcomes are described in Table 1. The mean age was 66.3 years; all patients were male, and 1329 (93%) patients were ASA class 1 or 2. During anesthesia, 970 mcg of remifentanil and 81 mg of rocuronium were used on average for a mean of 4.0 h. The mean postoperative hospital LOS was 7.8 days. Twenty-nine patients were unexpectedly readmitted to the hospital within 30 days postoperatively. The mean hospital charge was $2918.

**Table 1:** Characteristics and outcomes of patients who underwent robot-assisted laparoscopic prostatectomy

Variable	All patients (*N* = 1430)
Age, year	66.3 (7.2)
Body mass index, kg m^−2^	24.7 (5.1)
ASA physical status	
1	486 (34%)
2	843 (59%)
≥ 3	101 (7.1%)
Charlson Comorbidity Index score	4.73 (1.22)
2	21 (1.5%)
3	165 (11.5%)
4	456 (31.9%)
5	479 (33.5%)
6	202 (14.1%)
≥ 7	107 (7.7%)
Anesthesia related factors	
NMB reversal agents (sugammadex vs. neostigmine)	924 (64.6%) vs. 506 (35.4%)
Intraoperative remifentanil dose, per 100 mcg	9.7 (4.6)
Intraoperative rocuronium dose, per 10 mg	8.1 (2.2)
Intraoperative estimated blood loss, mL	166.1 (138.2)
Duration of anesthesia, h	4.0 (0.8)
Outcomes	
Length of hospital stay, days	7.8 (2.1)
Readmission within 30 days	29 (2%)
Unplanned readmission within 30 days due to surgical problem	18 (1.3%)
Cause of unplanned readmission within 30 days due to surgical problems	
Voiding difficulty	10/18 (56%)
Intrapelvic fluid collection	4/18 (22%)
Ileus	2/18 (11%)
Charge	
Total charges for postoperative hospitalization, United States dollars	11,034 (1942)
Net charges for postoperative hospitalization, United States dollars	2918 (1271)
Charges for anesthesia, United States dollars	344 (78)

*ASA* American Society of Anesthesiologists, *NMB* Neuromuscular blockade

Presented as n (%) or mean (standard deviation) or median (interquartile range)

Unplanned surgery-related readmission within 30 days occurred in 18 patients (1.3%) (Table 1). The most common causes of such readmissions were voiding difficulty requiring Foley insertion (10/18, 56%), intrapelvic fluid collection requiring percutaneous catheter drainage (4/18, 22%), and ileus (2/18, 11%).

Table 2 shows the pre-propensity score matching (sugammadex group: 924; neostigmine group: 506) and post-propensity score matching (sugammadex group: 444; neostigmine group: 444) covariate comparisons. After propensity score matching, all covariates were well-balanced (absolute standardized difference ≤ 0.1). The propensity score distribution became similar between groups after propensity score matching.

**Table 2:** Comparison between sugammadex and neostigmine groups before and after propensity score-matching. Presented as n (%) or mean (SD)

	Before propensity score-matching (n = 1430)		ASD	After propensity score-matching (*n* = 888)		ASD
	Sugammadex (*n* = 924)	Neostigmine (*n* = 506)		Sugammadex (*n* = 444)	Neostigmine (n = 444)	
Age, year (≥65)	579 (62.7)	310 (61.3)	0.03	270 (60.8)	261 (58.8)	0.04
Body mass index, kg m^−2^	24.5 (2.7)	25.1 (7.7)	0.20	24.7 (2.7)	24.9 (6.2)	0.08
Charlson Comorbidity Index score	4.7 (1.2)	4.8 (1.2)	0.07	4 .8 (1.3)	4.8 (1.2)	0.06
ASA physical status						
1	302 (32.6)	184 (36.4)		159 (35.8)	151 (34.0)	
2	554 (60.0)	289 (57.1)	0.05	255(57.4)	261 (58.8)	0.03
≥ 3	68 (7.4)	33 (6.5)	0.03	30 (6.8)	32 (7.2)	0.02
Intraoperative rocuronium dose, mg						
≥ 50, and < 100	742 (80.2)	420 (82.8)	0.02	381 (85.8)	383 (86.3)	0.01
≥ 100	176 (19.0)	78 (15.4)	0.11	32 (7.2)	39 (8.8)	0.05
Intraoperative remifentanil dose, per 100 mcg (≥10)	76 (8.2)	112 (22.1)	0.52	62(14.0)	59 (13.3)	0.02
Total intravenous anesthesia	68(13.4)	38(4.1)	0.47	35(7.9)	29(6.5)	0.07
Duration of anesthesia, h	3.9 (0.7)	4.2 (0.8)	0.34	4.1 (0.8)	4.1 (0.7)	0.07
Estimated blood loss, L	0.15 (0.1)	0.19 (0.1)	0.25	0.2 (0.2)	0.2 (0.1)	0.08
Distance, km^a^						
≥ 40, and < 180	44(8.7)	191(20.7)	0.29	44(9.9)	40(9.0)	0.02
180≥	20(2.0)	201(21.8)	0.43	20(4.5)	28(6.3)	0.04
Operation by skilled surgeon^b^	810 (87.6)	407 (80.3)	0.21	367 (82.7)	382 (860.)	0.10

*ASA* American Society of Anesthesiologists, *ASD* absolute value of standardized mean difference

Presented as n (%) or mean (standard deviation)

^a^ Distance means the distance between home and the hospital

^b^Surgeons who had done robot-assisted laparoscopic prostatectomy more than 200 cases

On a Poisson generalized linear model with a logarithmic link function using the propensity score-matched cohort, sugammadex use (vs. neostigmine) was associated with 6% decrease in hospital LOS (OR 0.94, 95% CI [0.89, 0.98], *P* = 0.008) and 8% decrease in post-anesthetic recovery time (OR 0.92, 95% CI [0.90, 0.94], *P* < 0.001); however, this did not reduce the 30-day unplanned readmission rate (Table 3; *P* = 0.288).

**Table 3:** Effect of sugammadex on length of stay in the post-anesthesia care unit, post-operative hospital stay and unplanned readmission, as compared to neostigmine, in patients who underwent robot-assisted laparoscopic prostatectomy, based on a propensity score-matched cohort

	Length of stay in the post-anesthesia care unit (min)			Hospital LOS after surgery (days)			Unplanned readmission within 30 days		
Variable	Mean (SD)	Odds ratio [95% CI]	*P* value^a^	Mean (SD)	Odds ratio [95% CI]	*P* value^b^	N (%)	Odds ratio (95% CI)	*P* value^c^
Sugammadex vs. Neostigmine	36.7 (8.4) vs. 40.2 (13.0)	0.92 [0.90, 0.94]	< 0.001	7.7 (2.5) vs. 8.2 (2.0)	0.94 [0.89, 0.98]	0.008	9 (2.0%) vs. 5 (1.1%)	1.82 [0.60,5.46]	0.288

^a,b^Length of stay in the post-anesthesia care unit, hospital length of stay after surgery: a generalized linear model assuming Poisson distribution and a log link function were used. *P* < 0.05 was considered as statistically significant

^c^30-Day unplanned readmission: logistic regression analysis was used. *P* < 0.05 was considered as statistically significant

On a gamma generalized linear model with a logarithmic link function with the propensity score-matched cohort, the anesthesia charge was increased (OR 1.07, 95% CI [1.04, 1.10], *P* < 0.001) in patients who received sugammadex, compared to those who received neostigmine. However, there were no significant differences between the groups as regards postoperative net charge (OR 1.04, 95% CI [1.00, 1.09], *P* = 0.061) and total charge (OR 0.98, 95% CI [0.96, 1.00], *P* = 0.100; Table 4).

**Table 4:** Effect of sugammadex on the charge for anesthesia, net charge, and total charge, as compared to neostigmine, in patients who underwent robot-assisted laparoscopic prostatectomy, based on a propensity score-matched cohort

Variable	Charge for anesthesia (USD)			Postoperative net charge^a^(USD)			Postoperative total charge(USD)		
	Median (IQR)	Odds ratio [95% CI]	P value^b^	Median (IQR)	Odds ratio (95% CI)	P value^c^	Median (IQR)	Odds ratio (95% CI)	P value^d^
Sugammadex vs. Neostigmine	343 (307–393) vs. 326 (291–361)	1.07 [1.04, 1.10]	< 0.001	2589 (2246–3347) vs. 2456 (2180–3179)	1.04 [1.00, 1.09]	0.061	10,875 (10055–11,843) vs. 11,588(9659–12,522)	0.98 [0.96, 1.00]	0.100

*IQR* interquartile range

^a^Net hospital charge: total hospital charge - charges for surgery and anaesthesia

^b,c,d^ Generalised linear model assuming gamma distribution and log link function was used, and *P* < 0.05 was considered as statistically significant

## Discussion

In this study, the reversal of NMB using sugammadex in RALP was shown to reduce the hospital LOS by 6% and decrease the post-anesthetic recovery time by 8%, compared to neostigmine; however, there was no impact on unplanned readmission within 30 days after the operation. The use of sugammadex had no effect on the net hospital charge and total charge after RALP, although we revealed that charge for anesthesia was increased.

Oh et al. similarly showed that sugammadex reduced hospital LOS; however, they found that it reduced net charge and 30-day unplanned readmission in patients who underwent major abdominal surgery [6]. In contrast, we found no reduction in unplanned readmission, even after considering the residual distance from the hospital (less than 50 km). This difference between the studies may be related to the different types of surgery between the studies. Oh et al. included study subjects who underwent major abdominal surgery with a procedure time > 2 h and estimated blood loss > 500 mL. On the other hand, RALP is a prolonged surgery, lasting 3.8 h, and involving the steep Trendelenburg position. However, most of the elective surgeries were performed by skillful expert surgeons, with a mean blood loss of only 166.1 mL, and the readmission rate was only 1.3% in our hospital.

We could not find appropriate and reliable records of pulmonary complications in patients who underwent RALP. Postoperative chest imaging or laboratory or device-based monitoring of oxygenation was not routinely performed after this surgery. Thus, we could not retrospectively assess the incidence of lung complications, such as atelectasis, bronchitis, pulmonary collapse due to mucus plugging of the airways, or pneumonia, related to surgery. Alternatively, we reviewed post-anesthetic recovery records, but there were no critical respiratory events by predefined definition [12] except the two patients who had cardiovascular events with known coronary artery disease. Therefore, we used postoperative recovery room LOS, hospital LOS, readmission rate, and net charges as surrogates [13–15].

For laparoscopic or robot-assisted surgery, the duration of the operation, the patient’s age, smoking status, and residual NMB, have been reported to be related to a prolonged hospital stay [16]. The prevalence of residual NMB in the post-anesthetic recovery room (TOF < 0.9) was reported to be about 64% in several multicenter studies. Residual NMB makes patients vulnerable to hypoxic damage and can cause aspiration due to the weakness of the upper airway muscle following increased recovery time and postoperative hospital stay [17]. This is consistent with our result since sugammadex reduced the LOS in the post-anesthetic recovery room and the hospital. According to a study by Murphy and colleagues, residual NMB caused a 150-min prolongation of mechanical ventilation duration in the intensive care unit in patients who had undergone cardiac surgery [6, 18, 19].

Sugammadex increased the anesthesia charge; however, it did not increase the postoperative net and total charges related to RALP. Although the use of sugammadex reduced the hospital LOS, it had no effect on the net hospital charge in our study. This result might be due to several reasons.

First, the net charge was defined at the total charge of healthcare services provided during admission except for the charge of the operation and anesthesia; thus, the staff expenses per time were excluded. There was a marked difference in concept between charge and cost. We analyzed the charge (i.e., the amount paid by the patient and government for our hospital’s medical services and medical products) because we could not obtain sufficient data to calculate cost retrospectively. Several review articles have also found that the use of sugammadex had no benefits on overall hospital costs [4, 20, 21]. In studies that claimed the cost-effectiveness of sugammadex, the “saved time” of anesthetic recovery was measured and multiplied with “the estimated value of the time of each staff member.” In this manner, they proved decreased time spent in the recovery room. However, the national healthcare system and the staff working patterns differ between studies, and thus, we should interpret the results considering certain conditions.

Second, RALP is a stereotypical surgery that would make no economic difference among patients. Patients who underwent RALP had a shorter recovery time and fewer complications compared to those who underwent retropubic radical prostatectomy [22]. In our study, the average hospital stay was 7.8 days (standard deviation, 2.5 days). Moreover, compared to open retropubic or laparoscopic surgery, RALP is associated with a lower mortality and transfusion rate [7] which would reduce the postoperative hospital stay. There was no significant difference in total charge, including the charge for surgery, between sugammadex use and neostigmine use, even though anesthesia charge in cases where sugammadex was used was higher than those in which neostigmine was used. This indicated that the effect of sugammadex cost on the total charge was limited.

Finally, medical resources in South Korea are quite inexpensive because of the wide national insurance coverage. The major part of the financial burden was the charge for robotic surgery, but this was excluded from our analysis. Therefore, reduced hospital stays had no effects on the net charge. Risk factors for an increased net charge were the total dose of rocuronium used, the duration of surgery or anesthesia, and the Charlson Comorbidity Index. It is thought that a delicate operation would take a marked amount of time and would demand much more postoperative care.

There were several limitations to our study. First of all, this was a single-center study, and cannot be fully representative. Second, this could be considered a historical cohort retrospective study, as it included data from 2012 to 2017. Sugammadex was introduced to our hospital in 2013, and its use started from 2014. After the introduction of sugammadex, anesthesiologists were able to select NMB reversal agents based on their preferences; after it was made available in our hospital, almost all anesthesiologists appeared to have a preference for sugammadex over neostigmine. However, when we performed the analysis to find factors including the time of surgery, that influences clinical outcomes such as LOS in the PACU, hospital, charges, unplanned re-admission rates, the timing of the surgery did not impact the results. This may be attributed to the fact that staff at our hospital had started RALP surgery long before the study period, and had already developed a protocol for this surgery and anesthesia; the process was therefore well established before initiation of the study. Therefore, we did not consider the time of surgery in the propensity score matching model. Third, we used the intraoperative rocuronium dose but did not include the degree of NMB (moderate or deep) in the analysis. We usually monitor NMB using a nerve stimulator (EZstim II, ES400, Life-Tech, Camarillo, CA, USA), TOF scan (idmed, Drager, Telford, PA, USA), or NMT module (Nihon Kohden, Shinjuku, Japan) depending on the anesthesiologist’s preference. However, this was not recorded in the medical records. The volume of sugammadex required differs according to the degree of NMB (at most 16 mg kg^− 1^), which affects cost-effectiveness [20]. Lastly, in our study, the intraoperative dose of rocuronium was higher in the sugammadex group than in the neostigmine group. Apparently, anesthesiologists use rocuronium freely when they plan to use sugammadex, or they prefer sugammadex over neostigmine when they use a higher dose of rocuronium; the benefits of deeper block of NMJ are controversial. In a meta-analysis of ten studies, there was a reduction in intraabdominal pressure (IAP) in three studies, and in the pain score after 24 h of surgery in two studies; however, in other two studies there were no differences in terms of the post-operative pain score and LOS among deep or moderate NMB groups [23]. In contrast, higher doses of rocuronium may be related to residual curarization and prolongation of stay in the PACU. We performed an analysis using a PSM model matched with multiple factors including the rocuronium dose, as described in the methods section; as a result, the stay in hospital and the PACU was shortened in the group receiving sugammadex compared to that receiving neostigmine. This did not lead to an increase in critical respiratory events in the recovery room.

## Conclusion

In this study, we showed that the length of hospital stay, as well as the length of the postoperative stay in the PACU, after RALP was shorter in patients in whom sugammadex, rather than neostigmine, was used for reversal of NMB. The net charge and unplanned readmission rate within 30 days after surgery showed no benefit in the sugammadex group as compared to the neostigmine group. Further studies should investigate the economic advantage or postoperative complications (acute and long term) of using sugammadex according to the type of surgery. If its economic effectiveness is clarified, sugammadex can be used routinely, with rare complications.

## Data Availability

The dataset used and analyzed during the current study is available from the corresponding author on reasonable request.

## Abbreviations

ASA: American Society of Anesthesiologists
LOS: Length of stay
NMB: Neuromuscular blockade
RALP: Robot-assisted laparoscopic prostatectomy
TOF: Train-of-four
